# Proteomic analyses of human islets reveal potential markers of **β** cell dysfunction during prediabetes

**DOI:** 10.1172/jci.insight.182135

**Published:** 2026-03-19

**Authors:** Chiara Maria Assunta Cefalo, Teresa Mezza, Giuseppe Quero, Sergio Alfieri, Donatella Lucchetti, Filomena Colella, Alessandro Sgambato, Wei-Jun Qian, Andrea Mari, Alfredo Pontecorvi, Andrea Giaccari, Rohit N. Kulkarni

**Affiliations:** 1 Dipartimento di Medicina e Chirurgia Traslazionale, Università Cattolica del Sacro Cuore, Rome, Italy.; 2 Unit of Endocrinology and Diabetology, Fondazione Policlinico Universitario Agostino Gemelli IRCCS, Rome, Italy.; 3 Pancreatic Surgery Unit, Pancreatic Advanced Research Center (CRMPG).; 4 Multiplex Spatial Profiling Facility, Fondazione Policlinico Universitario Agostino Gemelli IRCCS, Rome, Italy.; 5 Biological Sciences Division, Pacific Northwest National Laboratory, Richland, Washington, USA.; 6 Institute of Neuroscience, National Research Council, Padova, Italy.; 7 Section on Islet Cell and Regenerative Biology, Joslin Diabetes Center, Department of Medicine, Beth Israel Deaconess Medical Center, Harvard Medical School, Harvard Stem Cell Institute, Boston, Massachusetts, USA.

**Keywords:** Endocrinology, Metabolism, Diabetes, Islet cells, Proteomics

## Abstract

The mechanisms driving progressive β cell dysfunction in type 2 diabetes remain incompletely understood. This study aimed to identify pancreatic islet proteome changes that could predict diabetes onset. We isolated islets from individuals without diabetes undergoing partial pancreatectomy, previously characterized for glucose tolerance, insulin sensitivity, and insulin secretion, using laser capture microdissection, and analyzed them via high-performance liquid chromatography–mass spectrometry. Proteomic analysis revealed that individuals with impaired glucose tolerance (IGT) had reductions in proteins regulating glycolysis (PGK1, G3P), lipid metabolism (ACBP, ARF1), glucose transport (14-3-3B), and insulin secretion (STARD10, CAPDS) compared with normal glucose-tolerant (NGT) individuals. Additionally, IGT islets showed impaired expression of proteins involved in glucose- and incretin-stimulated insulin response (CREB1, IQGA1). Stratification by β cell glucose sensitivity (βGS) indicated that individuals with lower βGS exhibited reduced levels of insulin maturation (ERO1B) and antiapoptotic proteins (CASP8, PAK2, SKP1), along with increased SEL1L, a factor promoting endocrine precursor differentiation. These findings suggest that early defects in glucose metabolism and insulin secretion characterize IGT, while reduced βGS may trigger compensatory mechanisms, through enhanced β cell survival or neogenesis, to delay type 2 diabetes progression. Overall, proteomic alterations in prediabetic islets provide potential early predictive markers and targets for interventions aimed at preserving β cell function.

## Abstract 2

This study provides novel insights into the molecular alterations in pancreatic islets associated with early β-cell dysfunction in non-diabetic individuals. Islet proteome of subjects with IGT showed early defects in glucose metabolism and insulin secretion. However, a decrease in beta-cell glucose sensitivity GS may induce compensatory mechanisms to delay progression to overt type 2 diabetes.

## Introduction

Type 2 diabetes is a progressive disease associated with a gradual decline in the mass of functional β cells (1, 2). Although molecular and morphological alterations begin several years prior to diagnosis, the plasticity of islet cells indicates their ability to significantly influence the time course of the disease (3). The emerging consensus is that several mechanisms, including transdifferentiation among α cells (4) or duct cells (5), and the dedifferentiation of endocrine cells from an active secretory state to a nonsecretory quiescent state (6), all contribute to the pathogenesis of overt diabetes. We, along with others, have previously demonstrated that diverse stimuli such as pregnancy (7), or insulin resistance (8), prompt the endocrine pancreas to increase the islet cell volume as an adaptive regulatory response to increased insulin demand. Diminished β cell glucose sensitivity (βGS) hampers this compensatory response (9), potentially leading to early β cell failure and the hyperglycemia onset. However, the alterations in specific genes and signaling proteins that drive these processes in β cells remain unclear. The advent of global “-omics” technologies has enabled researchers to directly address such issues, leading to several studies examining the mRNA and protein content of pancreatic islets.

Several studies have demonstrated alterations in the protein expression of different tissues involved in the pathogenesis of diabetes (10, 11) in type 1 diabetes mellitus (12) as well as in mouse models of obesity or insulin resistance (13). For example, Lu et al. (14) reported a significant increase in proteins involved in pathways linked to protein biosynthesis and endoplasmic reticulum (ER) stress and a decrease in those regulating insulin processing/secretion, energy utilization, and metabolism in islets of muscle IGF-I receptor–lysine-arginine diabetic mice compared with those of WT mice. El Ouaamari et al. (15) described reduced expression of proteins controlling energy metabolism, oxidative phosphorylation, hormone processing, and secretory pathways in two mouse models of insulin resistance (*ob/ob* and high-fat feeding). More recently, proteome analysis of pancreatic islets isolated from 9 different models of spontaneous or genetically induced insulin resistance has revealed that proteins involved in islet mitochondrial function are positively associated with “healthy metabolic status” (16).

Transcriptomic and proteomic studies of human islets are generally challenging due to the limited availability of islets from living donors. Even when islets are available for studies the lack of associated clinical and/or laboratory phenotyping data on the individuals, coupled with the potentially large inherent interindividual variability in the pathogenesis of type 2 diabetes can limit the interpretation and implications of the data. For example, Slieker et al. (17) observed that individuals with altered glucose metabolism, when clustered by clinical and phenotypical characteristics, exhibited different proteomic and metabolomic profiles, and in particular, the “insulin resistant” cluster showed aberrant plasma levels of proteins involved in the intracellular PI3K/Akt pathway while the “obese cluster” showed elevated cytokines.

The mechanism(s) that underlie the pathophysiology of prediabetes are not fully understood. One opportunity to explore the alterations that occur in this state is by examining samples collected from individuals undergoing partial pancreatectomy for pancreatic tumors. Based on a number of genes differentially expressed in islets extracted from patients undergoing partial pancreatectomy, Solimena et al. (18) classified the individuals into four groups: normal glucose tolerant (NGT), impaired glucose tolerant (IGT), type 2 diabetes, and type 3c diabetes. In particular, they reported an early dysregulation of genes involved in key pathways in impaired glucose-tolerant compared with NGT individuals. However, the proteomic analyses, extending the transcriptomic data at the functional level, were limited to NGT and diabetic individuals. Finally, a recent study investigating the proteomic and metabolomic profiles of individuals with prediabetes, who either progressed to diabetes or reverted to normoglycemia within 1 year, identified 14 proteins, primarily involved in immune response and carbohydrate metabolism pathways, that showed significantly higher plasma concentrations in those who developed diabetes (19).

In the present study we aimed to characterize the islet proteome in samples collected from humans exhibiting different metabolic conditions. Specifically, we compared those with NGT to those with IGT and distinguished between individuals with preserved βGS and those with impaired βGS in order to identify proteins that could serve as predictive markers for diabetes progression.

## Results

### Study participants.

Twelve individuals without diabetes were enrolled, subjected to oral glucose tolerance test (OGTT) before surgery, and classified as NGT (*n* = 7) or IGT (*n* = 5). The 2 groups were matched for age and anthropometric characteristics. As expected, the IGT participants showed higher basal insulin values compared with NGT participants (NGT, 3.7 ± 2.1 mUI/mL vs. IGT, 10.1 ± 4.2 mUI/mL; *P* = 0.003). IGT participants showed a significant reduction in glucose uptake during hyperinsulinemic euglycemic clamp (expressed as M value) compared with NGT participants (NGT; 7.78 ± 0.91 mg·kg^–1^/min^–1^ vs. IGT: 5.07 ± 0.43 mg·kg^–1^/min^–1^; *P* = 0.01). Finally, evaluation of βGS during the mixed meal test (MMT) showed a significant decrease in the IGT participants compared with NGT participants (NGT: 204 ± 68.1 pmol·min^–1^m^–2^·mM^–1^ vs. IGT: 61.1 ± 40.7 pmol·min^–1^m^–2^·mM^–1^; *P* = 0.01). Clinical and metabolic characteristics of study participants classified based on glucose tolerance are summarized in Table 1.

To determine whether changes in the islet proteome would be better reflected by a functional measure of β cells we further classified the individuals based on the mean βGS. Thus, the NGT and IGT populations were subgrouped into (a) HIGH-βGS (n = 5) and (b) LOW-βGS (n = 7). According to the classification by glucose tolerance, 5 individuals in the HIGH-βGS group were determined to be NGT, while among the LOW-βGS group 2 individuals belonged to the NGT group and the others were in the IGT group. Thus, these classifications showed that not all individuals labeled as NGT belonged to the high βGS group, suggesting that glucose-based diagnosis of glucose intolerance does not appropriately reflect β cell functional capacity. Low-βGS individuals exhibited substantial lower mean M values but higher mean HbA1c levels compared with those with high βGS. Clinical and metabolic characteristics of study participants classified based on βGS are summarized in Table 2.

### Comparative qualitative proteomic analysis.

Proteomic analyses of islets revealed a list of 815 proteins identified with high confidence in our cohort of 12 individuals. A comparison of the proteins with the Islet Gene Atlas (20, 21), as expected, revealed that all individuals expressed pancreas and islet cell–specific proteins with high confidence (e.g., insulin [INS]; glucagon [GCG]; somatostatin [SMS]; regenerating islet-derived 1 α [REG1A]; trypsin-3 [TRY3]; protein disulfide isomerase [PDIA1]; pancreas/duodenum homeobox protein 1 [PDX1]; pancreastatin [CMGA]; secretogranin [CHGB]).

**Figure 1:**
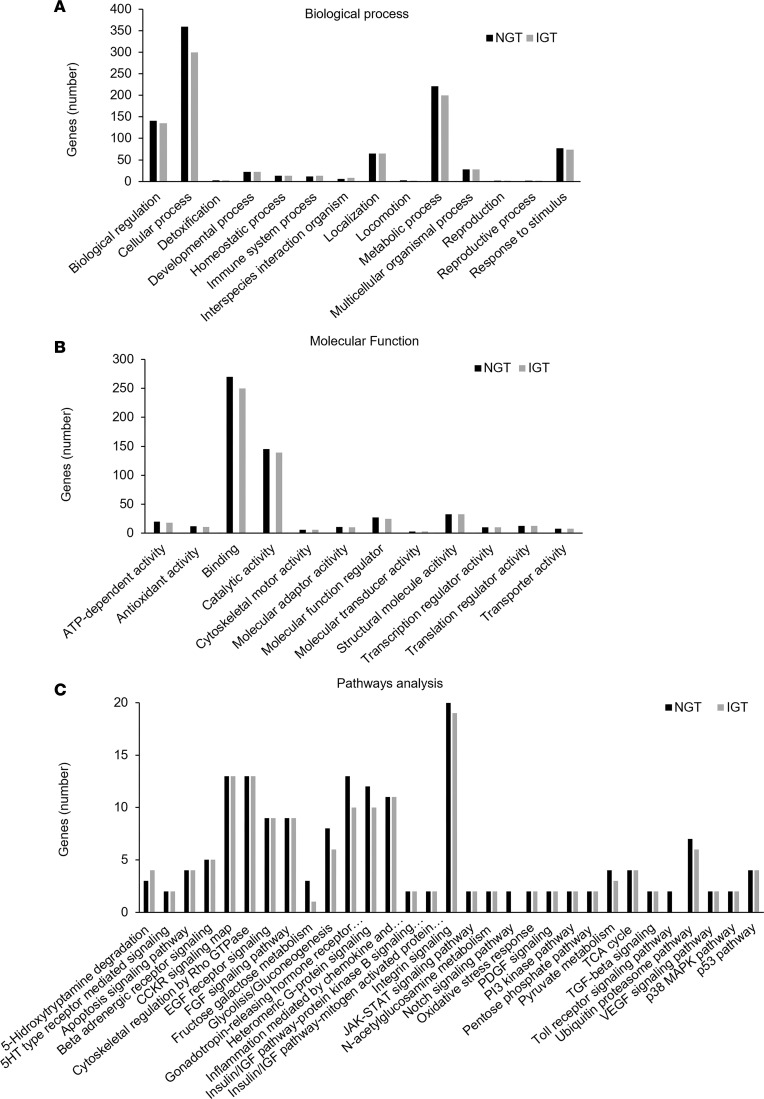
Gene ontology enrichment for differential genes between NGT islets and IGT islets. Categories were determined based on information provided by the online PANTHER classification system resource (https://pantherdb.org/validateHuman.jsp). All proteins were grouped based on biological process (**A**); molecular function (**B**); and pathways analysis (**C**). Data are expressed as the number of genes coding for the proteins identified in each group. NGT islets are represented as black bars, and IGT islets are represented as gray bars. In **C**, the underlying pathways represent those not expressed in IGT islets compared with NGT islets. NGT, normal glucose tolerant profile; IGT, impaired glucose tolerant profile.

In terms of biological functions, the majority of the proteins identified in both the NGT and IGT groups were involved in metabolic (glucose and lipid metabolism, metabolism of nuclear acids, etc.) and cellular (cell cycle and cell growth, signal transduction, intracellular and intercellular communication, etc.) processes. Interestingly, these proteins were reduced in islets from patients with IGT compared with those with NGT. Moreover, islets from patients with IGT had fewer proteins involved in biological regulation and response to stimulus compared with patients with NGT. Figure 1A shows the protein profile for each group, including the number of genes identified and classified by biological function.

Furthermore, we found that proteins involved in binding and catalytic activity were the most expressed in our cohort, with a reduced expression in IGT islets compared with NGT islets (Figure 1B).

Pathway analysis revealed loss of proteins involved in Toll-like receptors and notch signaling pathways in IGT islets. Moreover, IGT islets displayed a reduced expression of proteins involved in several key pathways, such as PI3 kinase, integrin signaling, G-protein signaling, or cell cycle; while an increased expression of proteins involved in 5-Hydroxytryptamine degradation pathway was detected in islets from patients with IGT compared with those with NGT (Figure 1C).

A striking observation when comparing the proteome of islets between the 2 groups revealed that several key proteins were not identified in islets from the IGT group compared with those from the NGT group, such as STARD10 and CADPS, which are involved in insulin granule biogenesis and secretion (22), or RAP1A, which is involved in glucagon-like peptide-1–linked (GLP-1–linked) regulation of insulin secretion (23). Furthermore, F262 and ALDOB, which participate in glycolysis, and ETFB, ATP5J, and SUCB1, which regulate ATP production through the Krebs’s cycle, were also not detected in the IGT proteome. Finally, ADAM10, a notch signaling pathway protein, regulating both cell fate and cell survival/growth (24) was identified in islets from patients with NGT but not in islets from patients with IGT.

Similar results were observed when comparing the islet proteome of individuals with high-βGS versus that from individuals with low-βGS (Supplemental Figure 1; supplemental material available online with this article; https://doi.org/10.1172/jci.insight.182135DS1).

### Pathways analysis of the differentially expressed proteins in islets from individuals with IGT compared with those with NGT.

Comparative quantitative analysis of islets, expressed as the log_2_ ratio of the average intensity of each individual protein, revealed 96 proteins differentially expressed in the IGT group compared with the NGT group (*P* value interaction between groups < 0.05), among which 51 were hyperexpressed and 45 hypoexpressed. We report all proteins expressed differently between the groups, with log_2_ of the average intensity and *P* value, in the Supplemental Table 1). A select group of regulated proteins implicated in different functional categories listed below in the Results were further examined.

**Figure 2:**
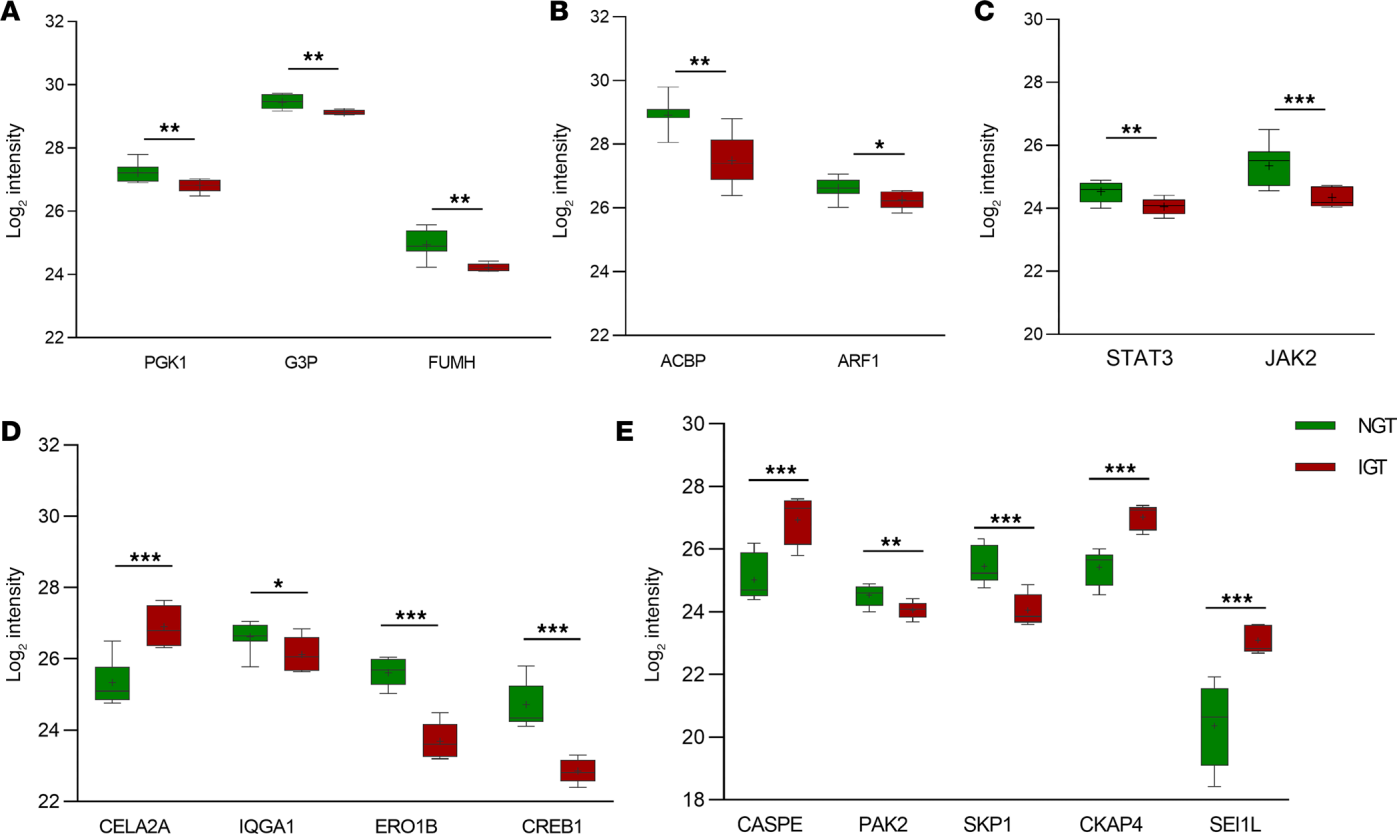
Differentially expressed proteins in IGT islets compared with NGT islets. Proteins involved in the major detected molecular pathways are grouped according to their function: glucose metabolism (**A**), lipid metabolism (**B**), intracellular signaling pathways (**C**), insulin secretion and release (**D**), or apoptosis and proliferation (**E**). IGT islets show increased levels of proteins involved in glycolysis (PGK1, G3P) and lower levels of proteins involved in the tricarboxylic acid cycle pathway (FUMH) and lipolysis (ARF1, ACBP) compared with NGT. IGT islets have impaired expression of proteins involved in the intracellular JAK/STAT signaling pathway (STAT3, JAK2). IGT islets have higher expression of proteins inducing insulin consumption (CEL2A) and lower expression of proteins essential for insulin cleavage (ERO1B) and insulin response to glucose (CREB1) and GLP-1 (IQAG1) stimuli compared with NGT islets. IGT islets reveal higher expression of protein with antiapoptotic function (CASPE) and lower expression of those with proapoptotic function (PAK2-SKP1) compared with NGT islets. IGT islets have increased levels of proteins inducing cell proliferation (CKAP4) and transdifferentiation from endocrine precursors (SEL1L) compared with NGT islets. NGT islets are represented as green boxes, and IGT islets are represented as red boxes. *P < 0.05; **P < 0.01; ***P < 0.001. IGT, impaired glucose tolerant (*n* = 5); NGT, normal glucose tolerant (*n* = 7).

Pathway analysis of the differentially expressed proteins in IGT islets compared with NGT islets revealed a decreasing expression of proteins involved in *gluconeogenesis* (PGK1, G3P); of proteins involved in the *tricarboxylic acid cycle pathway*, itself part of the carbohydrate metabolism (FUMH) (Figure 2A); and of proteins involved in *lipid metabolism pathway* (ACBP, ARF1) (Figure 2B). Moreover, there was a reduced expression of proteins involved in the *JAK-STAT signaling pathway* (STAT3, JAK2) (Figure 2C) which has been extensively related to β cells function in prediabetes conditions (25).

Proteins involved in *insulin processing pathway* were more highly expressed in islets from patients with IGT. For instance, CEL2A, an elastase known to stimulate insulin secretion and enhance insulin sensitivity (26), was present at higher levels in these islets (Figure 2D). Protein disulfide isomerases PDIA1 and PDIA4, which participate in converting preproinsulin to active insulin through reoxidation by ERO1B were increased (27), while ERO1B was reduced in IGT islets compared with NGT islets (Figure 2D). Additionally, islets from patients with IGT exhibited significantly lower levels of CREB1 (also known as the cAMP response element–binding protein), which regulates IRS2 gene expression in β cells upon glucose stimulation (28,29) (Figure 2D). Additionally, we observed a reduced expression of some proteins involved in *GLP-1 regulates insulin secretion pathways* (IQG1, PKA) in islets from patients with IGT compared with islets from patients with NGT (Figure 2D).

Proteins involved in *membrane trafficking pathway* were less expressed in IGT compared with NGT. The 14-3-3 family proteins, which are essential for GLUT4 translocation to the plasma membrane in response to insulin (30), were reduced in IGT islets compared with NGT islets.

Proteins involved in antiapoptotic function (e.g., CASPE, LMNA, LMNB2) were increased in IGT islets, while PAK2, an essential protein of *activated PAK-2p34 by proteasome mediated degradation* pathway with proapoptotic function, was reduced compared with NGT islets (Figure 2E). SKP1, an adaptor protein included in the ubiquitin ligase complex SCF (SKP1-CUL1-F-box protein) and known to participate in cell cycle progression, as well as having an important function in cell/cell interaction and communication (31), was significantly decreased in IGT islets compared with NGT islets (Figure 2E). Expression of proteins involved in the *insulin growth factor (IGF) signaling pathway*, known to mediate proliferation (ENPL, PDIA1, KLK1, CKAP4) (32, 33), was significantly higher in IGT islets (Figure 2E). Furthermore, we observed that expression of SEL-1 homologous protein1 (Sel1L), a component of the *ER-associated degradation (ERAD) pathway*, which has been previously reported to have a mitogenic action on β cells (34) and to induce differentiation of pancreatic precursors into the endocrine cell lineage (35), was higher in the IGT group compared with the NGT group (Figure 2E).

To ensure that the differential expression of proteins observed between IGT and NGT islets was not influenced by potential acinar contamination, suggested by the detection of proteins typically expressed in pancreatic acinar tissue (i.e., Chymotrypsin-like elastase family member, Pancreatic alpha-amylase, Carboxypeptidase A1) we performed a quantitative comparative analysis of the selected differentially expressed proteins, normalizing their levels to the expression of the acinar marker protein carboxypeptidase A1. As shown in supplemental materials, the differential expression patterns persisted after this correction, supporting the robustness of our findings (Supplemental Figure 2 and Supplemental Tables 2 and 3).

**Figure 3:**
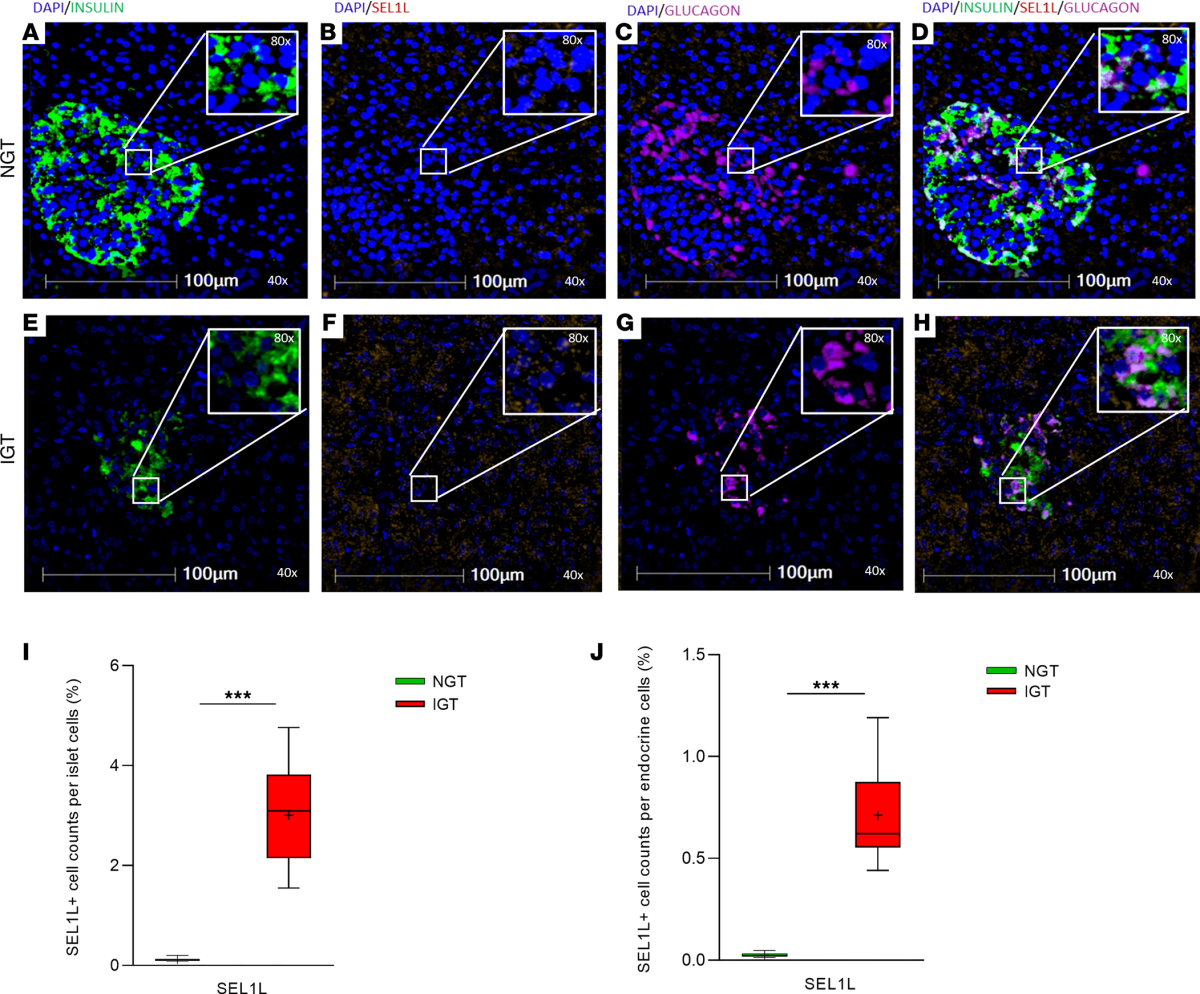
SEL1L expression in pancreatic samples from NGT and IGT individuals. Representative immunofluorescence images of pancreatic section from 1 individual with NGT (**A**–**D**) and 1 with IGT (**E**–**H**), stained for nuclei DAPI in blue (**A**–**H**), insulin in green (**A**–**E**), SEL1L in red (**B**–**F**), and glucagon in magenta (**C**–**G**). Merged images show the localization of SEL1L in relation to insulin- and glucagon^+^ cells in individuals with NGT (**D**) and IGT (**H**), respectively. SEL1L immunoreactivity appears higher in IGT islets compared with NGT islets. Scale bar: 100 μm. Original magnification, ×40; ×80 (insets). **I** showed the quantification of total SEL1L^+^ cells within islets, assessed across entire pancreatic sections (at least 10 islets per section) in individuals with NGT (green box) compared with those with IGT (red box). **J** showed the quantification of total SEL1L^+^ cells within exocrine cells, as assessed across entire pancreatic sections in individuals with NGT (green box) compared with those with IGT (red box). Data are expressed as mean ± SEM ***P < 0.01.

**Figure 4:**
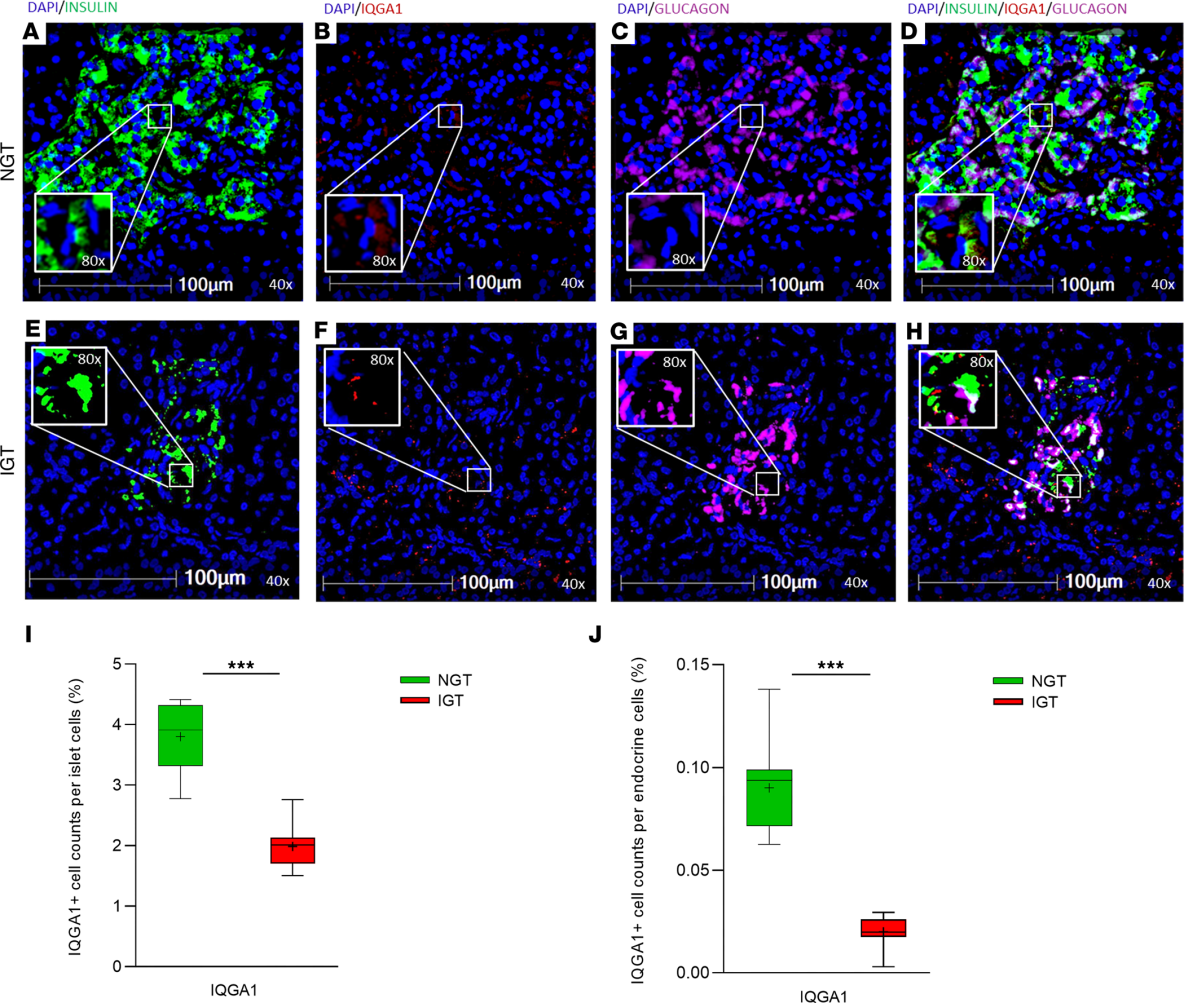
IQGA1 expression in pancreatic islets from patients with NGT and IGT. Representative immunofluorescence images of pancreatic section from 1 patient with NGT (**A**–**D**) and 1 with IGT (**E**–**H**), stained for nuclei DAPI in blue (**A**–**H**), insulin in green (**A**–**E**), and IQGA1 in red (**B**–**F**), and glucagon in pink (**C**–**G**). Merged images show the localization of IQGA1 in relation to insulin^+^ and glucagon^+^ cells in individuals with NGT (**D**) and IGT (**H**), respectively. IQGA1 immunoreactivity appears higher in IGT islets compared with NGT islets. Scale bar: 100 μm. Original magnification, ×40; ×80 (insets). **I** showed the quantification of total IQGA1^+^ cells within islets, assessed across entire pancreatic sections (at least 10 islets per section) in individuals with NGT (green box) compared with those with IGT (red box). **J** showed the quantification of total IQGA1^+^ cells within exocrine cells, assessed across entire pancreatic sections in individuals with NGT (green box) compared with those with IGT (red box). Data are expressed as mean ± SEM. ***P < 0.01.

To validate the differential protein expression identified in our proteomic analysis, we performed multiplex spatial imaging of 2 selected proteins, SEL1L and IQGA1, in representative pancreatic islet sections (NGT and IGT). To enable precise identification of islets within the pancreatic tissue, insulin and glucagon were included in the staining panel as specific markers of β and α cells, respectively. Tissue segmentation and quantitative analysis were carried out using HALO software, allowing for the localization and quantification of marker^+^ cells within both islets and exocrine regions. SEL1L^+^ cells were more frequently observed in IGT islets (Figure 3, E–H) compared with NGT islets (Figure 3, A–D), in agreement with the proteomic findings. Quantification, expressed as the percentage of SEL1L^+^ cells among total islet cells across at least 10 islets per individual, confirmed a higher proportion in the IGT group (Figure 3I). Notably, a higher proportion of SEL1L^+^ cells was detected in the exocrine compartment of the individuals with NGT compared with those with IGT (Figure 3J). In contrast, IQGA1^+^ was reduced in islets from the individual with IGT (Figure 4, E–H) relative to those from the individual with NGT (Figure 4, A–D), consistent with its downregulation in the proteomic dataset. A lower percentage of IQGA1^+^ cells within islets was observed in the IGT individual (Figure 4I), and a similar trend was noted in the exocrine tissue, with fewer IQGA1^+^ cells in the IGT versus the NGT individuals (Figure 4J). These findings independently validate our proteomic data and point to a potential role for SEL1L and IQGA1 in early islet dysfunction associated with prediabetes.

### Pathway analysis of differentially expressed proteins in individuals with altered βGS.

**Figure 5:**
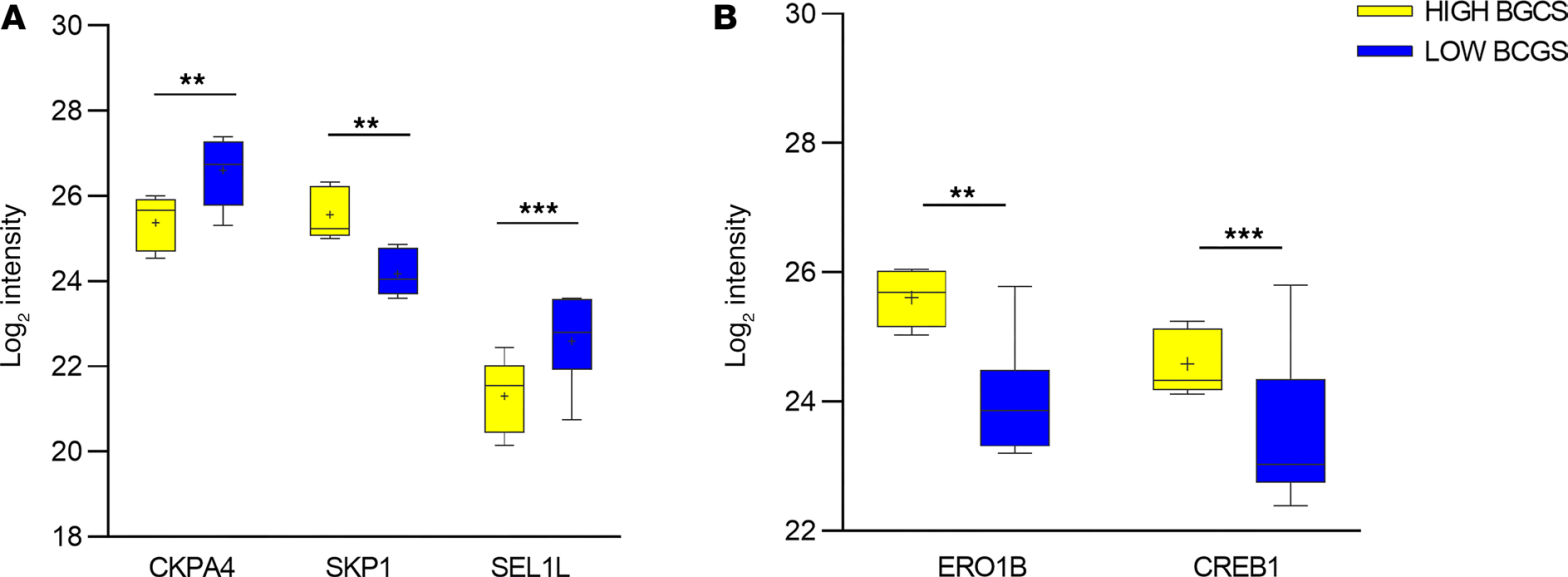
Differentially expressed proteins in the LOW-βGS group compared with HIGH-βGS group. Proteins involved in the major detected molecular pathway are grouped according to their function: apoptosis and proliferation (**A**) or insulin secretion and release (**B**). Islets of participants with LOW-βGS show increased levels of proteins involved cell proliferation (CKAP4) and transdifferentiation from endocrine precursors (SEL1L) compared with islets of participants with HIGH-βGS; however islets of individuals with LOW-βGS show reduced levels of proteins involved in apoptosis (SKP1), insulin maturation (ERO1B), and cell response to glucose stimuli (CREB1). The LOW-βGS group is represented as blue boxes, and the HIGH-βGS group is represented as yellow boxes. *P = interaction between HIGH-βGS vs. LOW-βGS. **P < 0.01, ***P < 0.001. βGS, β cell glucose sensitivity.

The comparative quantitative analysis revealed a total of 37 proteins differentially expressed in the islets of LOW-βGS individuals compared with those of HIGH-βGS individuals, among which 18 were hyperexpressed and 19 hypoexpressed. Notably, proteins involved in the *IGF1 signaling pathway,* such as CKAP4, which mediates cellular proliferation, were more highly expressed in LOW-βGS individuals compared with HIGH-βGS individuals. Moreover, proteins involved in *proapoptotic signaling pathways* (PAK2 and SKP1) were increased while a protein involved in the *antiapoptotic signaling pathway* (CASPE) was decreased in the LOW-βGS group compared with the HIGH-βGS group (Figure 5A). Interestingly, SEL1L, a protein of the *ERAD pathway*, which influences endocrine precursor differentiation under stress conditions, was more highly expressed in LOW-βGS individuals compared with HIGH-βGS individuals (Figure 5A). Furthermore, proteins crucial for insulin maturation (ERO1B) and glucose-stimulated β cell responses (CREB1) were diminished in LOW-βGS individuals (Figure 5B). These observations suggest that the reduction of βGS in individuals defined as NGT according to the commonly used classification leads to initial defects in insulin synthesis and triggers potential compensatory mechanisms to delay diabetes progression. However, alterations in proteins related to impaired glucose metabolism, lipid metabolism, and reduced insulin response to incretin stimulation were observed exclusively in individuals with IGT.

### Correlation between in vivo functional features and protein expression.

To identify the potential molecular “triggers” of islet plasticity in prediabetes we investigated correlations between metabolic functional data derived from in vivo studies (e.g., glucose tolerance, insulin resistance, insulin secretion, β cell function) and the expression of proteins of interest in our study cohort.

**Figure 6:**
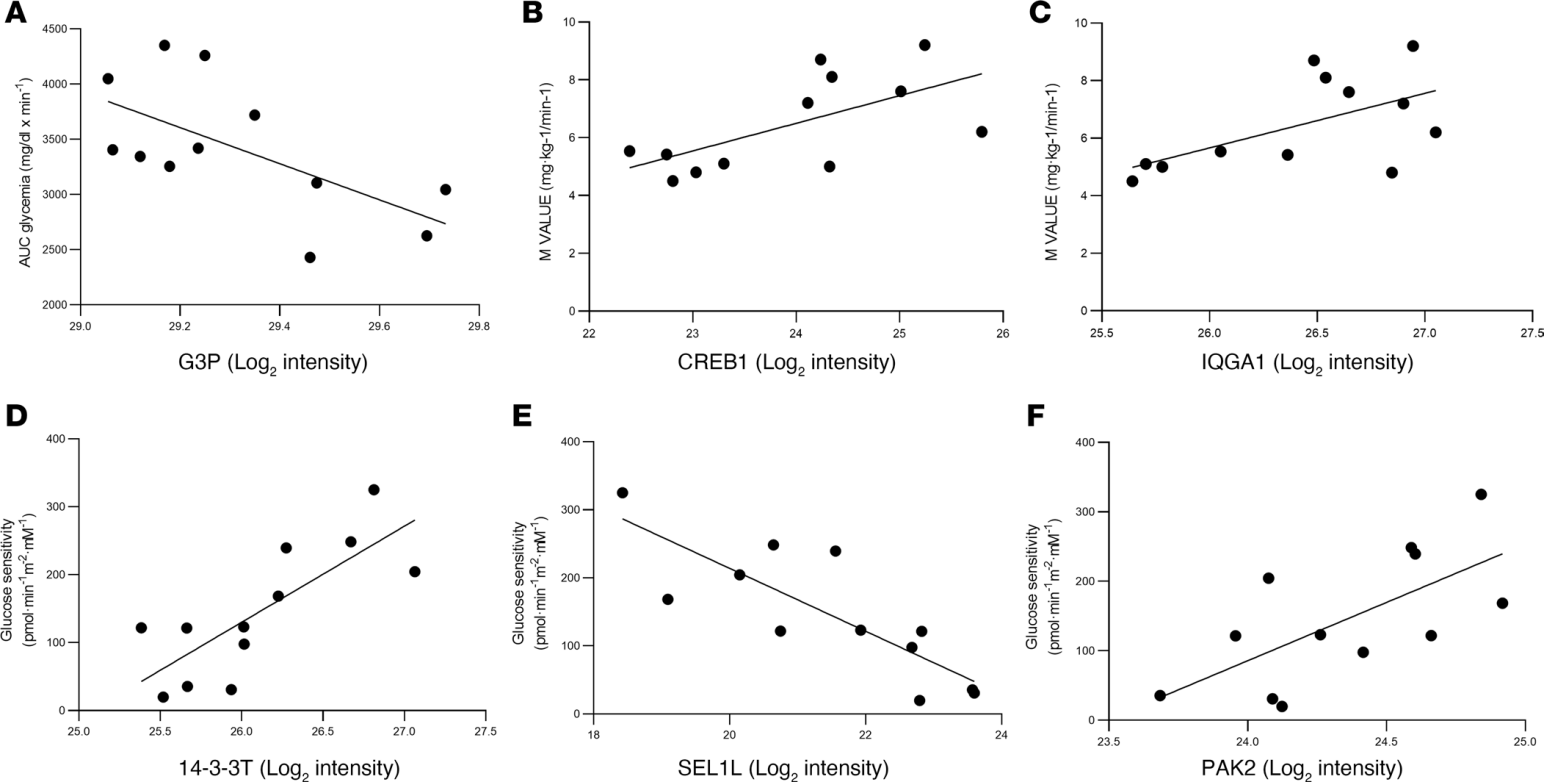
Correlation between expression of selected proteins and metabolic features of individuals with NGT versus those with IGT in vivo. In **A**, glucose tolerance is inversely correlated to expression of proteins involved in glycolysis (G3P); in **B**, insulin resistance (measured as M value during a hyperinsulinemic euglycemic clamp) is directly correlated to expression of proteins regulating insulin secretion upon glucose stimulus (CREB1); in **C**, insulin resistance (express as M value during a hyperinsulinemic euglycemic clamp) is inversely correlated to the expression of proteins involved in GLP-1–induced insulin production (IQGA1); in **D**, β cell glucose sensitivity is directly correlated to a protein involved in glucose homeostasis in response to insulin stimulus (14-3-3T); and in **E**, glucose sensitivity is inversely correlated to the expression of a protein increasing endocrine precursors differentiation (SEL1L) and directly correlated to a protein with reducing apoptosis function (PAK2). AUC glycemia, area under the curve of glucose.

First, we observed a significant inverse correlation between the glycemic response to oral glucose load, AUC of glucose, and expression of G3P, a protein involved in glucose metabolism (*r* = –0.63 [95% CI, –0.8842 to –0.08761]; *P* = 0.02) (Figure 6A). Second, insulin resistance, expressed as an M value during a hyperinsulinemic euglycemic clamp, was directly correlated with expression of both CREB1 (*r* = 0.6342, [95% CI, 0.09480 to 0.8857]; *P* = 0.02), a protein involved in insulin release upon glucose stimulus (Figure 6B), and IQGA1 (*r* = 0.58 [CI, 0.1688 to 0.8677]; *P* = 0.04), a protein involved in GLP-1–induced insulin production (Figure 6C). Third, we found a strong correlation between expression of 14-3-3T, a protein involved in translocation of GLUT4 to the plasma membrane, and βGS calculated during MMT (*r* = 0.78 [CI, 0.3784 to 0.9360]; *P* = 0.002) (Figure 6D). Finally, SEL1L was inversely correlated with βGS (*r* = –0.82 [CI, –0.9492 to –0.4757]; *P* = 0.001) while expression of PAK2, a proapoptotic factor, was directly correlated with βGS (*r* = 0.66 [CI, 0.1496 to 0.8972]; *P* = 0.01) (Figure 6, E and F). In addition, other proteins that were differentially expressed between NGT and IGT individuals showed marked correlations with derived indices of β cell function, including homeostasis model of β cell function and the disposition index (Supplemental Tables 2 and 3). These data suggest that changes in the expression of proteins involved in islet compensative mechanisms directly reflect in vivo alterations of β cell function, insulin secretory capacity, and the consequent impairment in glycemic control.

## Discussion

Our study revealed specific alterations involving β cell responsiveness, insulin secretory capacity, and β cell fate in the islet proteome of nondiabetic humans. Interestingly, we conducted a comprehensive proteomic analysis of human islets obtained from patients phenotyped for insulin secretion capacity and insulin sensitivity, enabling correlation of in vivo metabolic features with the proteomic profile of islets. Furthermore, a comparative study of islets from nondiabetic and IGT individuals allowed us to gain insights into the early phases of the natural history of type 2 diabetes and to identify potential predictors of disease progression.

We classified study patients based on clinical history and HbA1c in addition to the parameters that are considered gold standards in glucose homeostasis (e.g., OGTT, MMT, and euglycemic hyperinsulinemic clamp). This allowed us to determine insulin secretion, insulin resistance, and measures of β cell function in vivo. Furthermore, as reported previously in studies investigating changes in insulin secretory capacity during the natural history of both type 1 and type 2 diabetes (36–38), we adopted βGS as a unique measure of β cell function in vivo. The findings from the comparative analyses of islets from patients with IGT versus patients with NGT were similar to those of previous reports (14, 17). For example, in islets from the IGT group we observed a substantial reduction of proteins involved in several metabolic processes, such as glucose metabolism (PGK1, GP3, F262 and ALDOB), lipid metabolism (ARF1, ACBP), and alternative energy metabolisms (FUMH) (15, 18). Thus, while these results are consistent with those of previous reports they also provide potentially novel insights.

It has been speculated that the IGT state is characterized by a reduced capability to adequately respond to both glucose (39, 40) and incretin stimulation (41, 42). Consistently, in islets from patients with IGT, we observed a reduction in the expression of CREB1, a protein reported to regulate IRS2 to modulate efficient glucose sensing, insulin exocytosis, insulin gene transcription, and β cell survival in response to glucose or incretin stimulation (28, 29). However, contrary to previous findings reporting an attenuated expression of IRS2 in islets of patients with longstanding type 2 diabetes, we observed its protein expression to be comparable between NGT and IGT islets. We also observed that IQGA1, a protein involved in the regulation of insulin secretion mediated by GLP-1, was reduced in islets from patients with IGT, suggesting an impairment in incretin sensitivity in response to insulin resistance since its expression is directly associated with worsening insulin sensitivity. In addition, the lack of expression of RAP1a in islets from patients with IGT, a Ras-related GTPase-mediating Epac2 activation, which leads to GSIS potentiation through GLP-1 signaling (23), underscored the reduced responsiveness of the β cell to the incretin hormone as a potentially early defect in prediabetes.

In addition to altered sensing, it is known that IGT is directly linked to altered insulin secretory capacity. In the islets from patients with IGT, compared with those from patients with NGT, we detected a decrease in ERO1b, a protein involved in insulin maturation, while we found no expression of both STARD10 and CADPS, proteins involved in insulin release from granules, in patients with IGT. Previous studies have demonstrated that reduced STARD10 expression is associated with impaired glucose-stimulated Ca^2+^ dynamics, insulin secretion (22), and impaired insulin granule ultrastructure (44). Deletion of CAPDS, on the other hand, correlates with decrease in size and number of the pool of secretory insulin vesicles, slowing of granule release, and suppression of second-phase insulin secretion (45). This finding is consistent with our previous study in a model of acute β cell mass reduction where we observed that the preservation of rate sensitivity in the presence of decreased βGS slows progression from IGT to overt type 2 diabetes (3).

A comparison between the protein profile of islets from patients with NGT versus patients with IGT revealed significant differences in the expression of proteins regulating cell fate. Specifically, we observed that islets from patients with IGT displayed an increase in antiapoptotic factors coupled with a decrease in proteins involved in proapoptotic function, suggesting that an overall decrease in apoptosis contributes to preserving β cell functional mass. Although apoptosis has been described as one of the major mechanisms leading to β cell loss during the natural history of type 2 diabetes (45, 46), studies from our group and others (8, 9) have failed to document an increase in β cell apoptosis in response to insulin resistance. It is possible that apoptosis is unmasked only during final stages of the disease.

Some studies have argued that ER stress is an important determinant of β cell dysfunction in the pathogenesis of both type 1 and type 2 diabetes (35, 36). Consistently, we observed that islets from patients with IGT expressed higher levels of SEL1l protein, a component of the ERAD pathway, which has been reported to induce differentiation from endocrine precursors to mature endocrine cells (34, 35, 47). Finally, lack of expression of ADAM10 in islets from patients with IGT could imply that it has an important role in the regulation of islet cell fate at this stage. Over the past two decades, numerous reports have provided evidence of the pivotal role of Notch signaling in pancreatic cell proliferation, differentiation, and plasticity (48, 49), and inhibition of this pathway has been reported to cause premature differentiation of the multipotent progenitor cells into endocrine cells (50) and to promote acinar cell differentiation (51). Thus, changes in the expression of proteins involved in Notch signaling could indicate an attempt to preserve a greater number of endocrine cells in their mature and metabolically active state, prior to a loss of the β cell phenotype and progression to overt diabetes.

The development of overt diabetes has traditionally considered the progression from NGT to IGT. We took advantage of our classification based on βGS (a measure of secreted insulin in response to a specific glucose concentration) (52) to interpret alterations in the islet proteome in these patients. Guided by changes in βGS we observed that β cell dysfunction is linked to a progressive reduction of ERO1B expression, an ER stress protein regulating preproinsulin maturation to an active insulin form, suggesting impaired insulin synthesis in individuals with blunted βGS (36. These findings are consistent with those in our previous report that insulin-resistant nondiabetic patients already exhibit an increased circulating proinsulin/insulin ratio, indicating that a defect in the insulin secretory machinery is already present in prediabetes (53). A second feature associated with the decrease in βGS was a concomitant decrease in antiapoptotic factors (PAK2 and SKP1) and an increase in SEL1l expression, supporting the notion that neogenesis of β cells and a reduction in the apoptosis of existing β cells both likely contribute to maintain endocrine cell functional mass to delay progression to type 2 diabetes (37).

Despite the unique approach of this study that allowed correlating the function of the endocrine pancreas in vivo with the islet proteome in early altered metabolic conditions, some limitations deserve comment. First, we were able to identify only a limited number of proteins in our samples. It is important to recognize that all the individuals included were nondiabetic, and no previous study to our knowledge has specifically focused on the prediabetic state. Second, although we used laser capture microdissection to avoid exocrine contamination, we cannot completely rule out sampling artifacts. Additionally, the study population consisted of White adult men and women recruited at a single institution; thus, the generalizability of the results to other ethnic groups remains to be established.

In sum, we suggest that poor glucose sensitivity is a potential trigger of endocrine cell transdifferentiation in an attempt by β cells to cope with higher demands for insulin secretion. Among the various compensation mechanism(s) during the prediabetes state, it is likely that regeneration from islet progenitors contributes to new β cells in individuals with poor βGS. Collectively, these results underscore the plasticity of human islet cells in response to stress or increased metabolic demand and proteins as early markers of β cell dysfunction that may be harnessed as potential therapeutic targets to delay progression toward type 2 diabetes.

## Methods

### Sex as a biological variable.

Our study examined male and female individuals, and similar findings are reported for both sexes.

### Patients, metabolic studies, and surgical procedures.

Participants — aged 18–75 years with no history of diabetes mellitus and candidates for pylorus-preserving pancreatoduodenectomy for nonmalignant pancreatic tumors —were recruited at the Digestive Surgery Unit and studied at the Centre for Endocrine and Metabolic Diseases unit (Agostino Gemelli University Hospital). Among the 12 enrolled participants, 5 had been diagnosed with intra-ampullary papillary-tubular neoplasm, 3 with the intraductal papillary mucinous neoplasm, 1 with serous cystic neoplasm, while 3 were reported to have a neuroendocrine tumor. Only eligible patients were subjected to a presurgery metabolic evaluation, which consisted of an OGTT, a MMT, and euglycemic hyperinsulinemic clamp for the evaluation of insulin sensitivity. For detailed methods regarding metabolic screening and surgical procedures please refer to the Supplemental Methods.

### Calculations.

During OGTT and MMT insulin secretion was derived from C-peptide levels by deconvolution (52). An estimate of the β cell function (defined as βGS) was obtained as an increase, compared with the basal value, of insulin secretion during the last 20 minutes of the test, divided by the corresponding increase in blood glucose as previously described (9, 52). Rate sensitivity, also estimated from OGTT modeling, is a β cell–functional parameter that represents the dependence of the ISR on the rate of change in glucose concentration and is related to early insulin release. Matsuda indices (54) provided a measure of whole-body insulin sensitivity based on insulin and glucose values obtained from the OGTT.

### Sample processing and proteomic analysis.

Pancreatic tissue samples extracted during the surgery at Agostino Gemelli University Hospital were placed in cryomolds, placed in OCT and rapidly frozen, and stored at –80°C until sectioned at the Core Facility at Joslin Diabetes Center, Harvard Medical School. All pancreas sections were immunostained with H&E and examined independently by 2 pathologists to exclude tumor infiltration prior to subsequent analyses. Subsequently, laser capture microdissection was carried out on these sections to isolate islets as previously described (55). The microdissected cells were then incubated with 15 μL of an elution/digestion buffer (8 mM NH_4_HCO_3_; 10 mM DTT; 50 Mm Trypsin pH 8) for 15 minutes at 37°C for protein digestion. The digestion was stopped by 0.1% TFA. All peptide samples were dried in a Speed Vac to remove TFA and resuspended in 20 mM NH_4_HCO_3_ for subsequent proteomic analysis. High-performance liquid chromatography-mass spectrometry analyses were performed at the Pacific Northwest Laboratories on a custom-built automated LC system coupled online to an LTQ-Orbitrap mass spectrometer (Thermo Scientific) via a nanoelectrospray ionization interface as previously described (56). The resultant raw data were converted into data files using Extract_MSn (version 3.0) in Bioworks Cluster 3.2 (Thermo Fisher Scientific). The analysis of the raw data was performed through a client-server application (Proteome Discovered version 2.0 produced by Thermo Fisher) able to identify different proteins by comparing the mass spectra of the digested fragments with the information contained in a selected FASTA database. We used the UniProt service, which collects information from the widely used Swiss-prot, TrEMBL, and PIR databases (57), to clarify the function and characteristics of each protein. Using Protein Analysis Through Evolutionary Relationships (PANTHER) classification software we classified the proteins (and their coding genes) expressed in each single group under study on the basis of their biological and molecular function and cell pathway (58, 59). Gene ontology was used to investigate protein functions. A detailed description of proteomic analysis is reported in the Supplemental Methods.

### Multiplex spatial imaging.

A 4-plex fluorescence panel was performed on 3 μm sections of FFPE human pancreatic tissue using antibodies against SEL1L, IQGAP1, insulin, and glucagon. Immunostaining was carried out on a Leica BOND RX automated immunostainer (Leica Microsystems) using an Opal IHC kit (PerkinElmer; NEL820001KT). Fluorophores and DAPI were prepared according to the manufacturer’s instructions. Supplemental Table 4 provides detailed information on the antibodies, including dilutions and retrieval buffers. The PhenoImager HT 2.0 system (Akoya Biosciences) was used to acquire images with spectral unmixing at ×20 magnification, followed by processing with HALO software (Indica Labs LLC) (60). Those experiments were performed at Multiplex Spatial Profiling Facility (Agostino Gemelli University Hospital).

### Statistics.

All data are expressed as mean ± SEM, except where otherwise indicated. Differences between means were tested using Student’s 2-tailed *t* test. The relationships between different variables were derived by linear regression analysis using Graphpad Prism (version 6). Regarding the clinical characteristics of the patients and the correlations between in vivo parameters and protein expression, a *P* value less than 0.05 was considered statistically significant. In the comparative proteomic analysis between groups, as previously reported (55), only proteins with the following criteria were considered statistically significant: *P* value less than 0.05 and a ratio of log_2_ of the mean intensity between 2 compared groups <–0.58 or >0.58.

### Study approval.

The study protocol (ClinicalTrials.gov NCT02175459) was approved by the local ethics committee, CET territoriale Roma 3 (P/656/CE2010 and 22573/14) (Rome, Italy), and all participants provided written informed consent prior to all study procedures.

### Data availability.

Supporting data values for all figures and supplemental figures are provided in the Supporting Data Values file. Additional data are available from the corresponding author upon reasonable request. Deidentified human subject data can be shared, where permitted, in accordance with institutional and ethical guidelines.

## Author contributions

CMAC and TM collected and analyzed data and equally contributed to write the manuscript. GQ, AM, and WJQ collected and analyzed data. DL, FC, and AS performed and analyzed data from multiplex spatial imaging. SA and AP edited the manuscript. AG and RNK designed the study, interpreted data, and reviewed the manuscript. All authors have read and agreed to the published version of the manuscript.

## Conflict of interest

RNK is on the scientific advisory boards of Novo Nordisk, Biomea, and REDD.

## Funding support

This work is the result of NIH funding, in whole or in part, and is subject to the NIH Public Access Policy. Through acceptance of this federal funding, the NIH has been given a right to make the work publicly available in PubMed Central.

- NIH (R01DK67536, R01HD105947, and R01DK132900 to RNK).
- NIH (P30DK036836 to the Joslin Diabetes Research Center).
- Università Cattolica del Sacro Cuore (Fondi Ateneo Linea D.3.2 to TM).
- Ministero dell’Istruzione, dell’Università e della Ricerca (PRIN 2015373Z39_006 to TM).
- Ministero della Salute (GR-2018-12365577 to TM).
- Fondazione Diabete Ricerca (FODIRI-MSD 2014/2015 to CMAC).
- European Association for the Study of Diabetes (Albert Reynolds Travel Fellowship) to CMAC.
- Diabetes Wellness Research Foundation Chair to RNK.

## Supplementary Material

Supplemental data

Supporting data values

## Figures and Tables

**Table 1:**
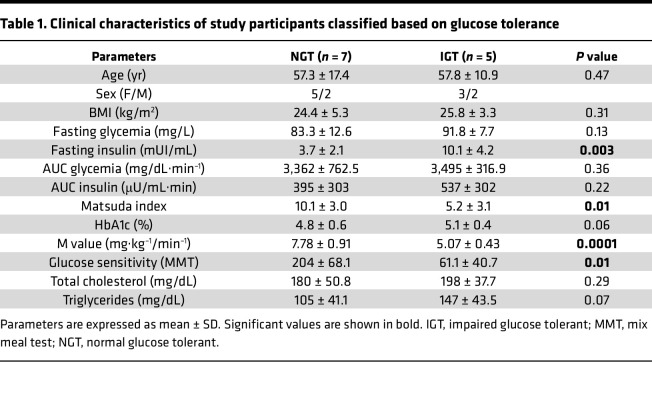
Clinical characteristics of study participants classified based on glucose tolerance

**Table 2:**
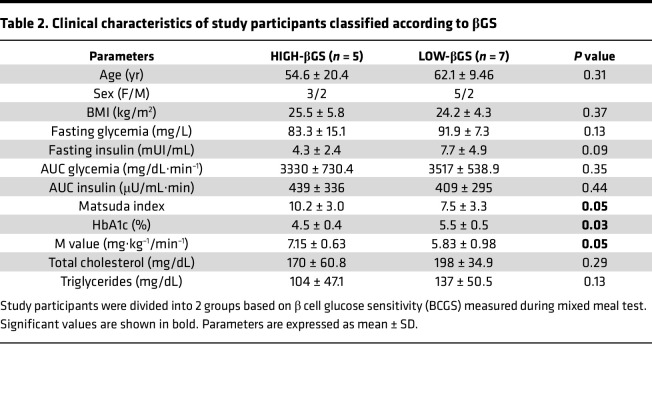
Clinical characteristics of study participants classified according to βGS

## Version 1 03/19/2026

In-Press Preview

## Version 2 05/08/2026

Electronic publication
