# Supplementary material for: Proteomic analyses of human islets reveal potential markers of β cell dysfunction during prediabetes
Source: JCI Insight. 2026 Mar 19;11(9):e182135. doi: 10.1172/jci.insight.182135 (PMC13232019; doi:10.1172/jci.insight.182135)
Supplement: Supplemental data [file jciinsight-11-182135-s123.pdf]

ELECTRONIC SUPPLEMENTARY MATERIALS

RESULTS

Tab.S1 Proteins differentially expressed in IGT islets compared to NGT ones.

GENE	PROTEIN NAME	Fold difference	p-value (IGT vs NGT)
**DOWN-REGULATED**			
ERO1B_HUMAN	ERO1-like protein beta	-1,953844908	0,02181316
ACBP_HUMAN	Acyl-CoA-binding protein	-1,702970609	0,013691528
K7EJ78_HUMAN	40S ribosomal protein S15	-1,560678607	0,02310156
XRCC5_HUMAN	X-ray repair cross-complementing protein 5	-1,547164203	0,00978991
RTN1_HUMAN	Reticulon-1	-1,497253531	0,000653076
E9PM92_HUMAN	Chromosome 11 open reading frame 58	-1,345711948	0,029607711
CREB1_HUMAN	Cyclic AMP-responsive element-binding protein 1	-1,292260173	0,000472191
S10A6_HUMAN	Protein S100-A6	-1,276943359	0,03883882
LAMB1_HUMAN	Laminin subunit beta-1	-1,20890494	0,008559424
SERPH_HUMAN	Serpin H1	-1,167125471	0,012797491
AMD_HUMAN	Peptidyl-glycine α-amidating monooxygenase	-1,147496275	0,035155488
E9PLF1_HUMAN	Glutathione S-transferase Mu 2	-1,141919644	0,024770904
SKP1_HUMAN	S-phase kinase-associated protein 1	-1,138526642	0,032115795
RBM8A_HUMAN	RNA-binding protein 8A	-1,021146535	0,000262142
CMGA_HUMAN	Chromogranin-A	-0,900305985	0,018195622
MYH11_HUMAN	Myosin-11	-0,833387402	0,007511186
GORS2_HUMAN	Golgi reassembly-stacking protein 2	-0,740330234	0,012289888
PURA_HUMAN	Transcriptional activator protein Pur-alpha	-0,67948095	0,011959482
CALM_HUMAN	Calmodulin-1	-0,673214077	0,004561325
KCY_HUMAN	UMP-CMP kinase	-0,621369024	0,007840769
UBA1_HUMAN	Ubiquitin-like modifier-activating enzyme 1	-0,600638412	0,041482441
1433T_HUMAN	14-3-3 protein theta	-0,588532808	0,034623284
IGHG1_HUMAN	Immunoglobulin heavy constant gamma 1	-0,579460639	0,009075145
HNRPL_HUMAN	Heterogeneous nuclear ribonucleoprotein L	-0,576469862	0,037886635
Q5JRI1_HUMAN	Serine/arginine-rich-splicing factor 10	-0,542519888	0,007747543
A0A0B4J1Z1_HUMAN	Serine/arginine-rich-splicing factor 7	-0,532427776	0,009851968
COPB2_HUMAN	Coatomer subunit beta	-0,52123758	0,019968621
JAK2_HUMAN	Tyrosine-protein kinase JAK2	-0,517356548	0,00649759
PAK2_HUMAN	Serine/threonine-protein kinase PAK 2	-0,5105904	0,012087313
C9JZG1_HUMAN	Eukaryotic translation initiation factor 3 sub.B	-0,507389815	0,011895173
SRSF1_HUMAN	Serine/arginine-rich splicing factor 1	-0,495926025	0,014324501
FUMH_HUMAN	Fumarate hydratase, mitochondrial	-0,486356509	0,024933355
VDAC1_HUMAN	Voltage-dependent anion-channel protein 1	-0,477973204	0,035530303

PPIA_HUMAN	Peptidyl-prolyl cis-trans isomerase A	-0,471334968	0,034582023
STAT3_HUMAN	Signal transducer and activator of transcription 3	-0,468003541	0,022613753
PGK1_HUMAN	Phosphoglycerate kinase 1	-0,406550442	0,022776397
F8W726_HUMAN	Ubiquitin-associated protein 2-like	-0,405857096	0,028686218
ARF1_HUMAN	ADP-ribosylation factor 1	-0,402031906	0,045349475
TCPG_HUMAN	T-complex protein 1	-0,392093655	0,007642673
IQGA1_HUMAN	Ras GTPase-activating-like protein	-0,371292868	0,015370799
ROA3_HUMAN	Heterogeneous nuclear ribonucleoprotein A3	-0,35391137	0,04782972
B8ZZC5_HUMAN	Glutaminase kidney isoform, mitochondrial	-0,34644975	0,030085921
G3P_HUMAN	Glyceraldehyde-3-phosphate dehydrogenase	-0,316013294	0,00674515
A0A075B6N8_HUMAN	Immunoglobulin heavy constant gamma 3	-0,298546989	0,04439445
PSA3_HUMAN	Proteasome subunit alpha type-3	-0,274085934	0,03844112
**UP-REGULATED**			
CASPE_HUMAN	Caspase-14	1,904031155	0,001866081
ERP27_HUMAN	Endoplasmicreticulumresidentprotein 27	1,780125812	0,00122635
RRBP1_HUMAN	Ribosome-bindingprotein 1	1,748101282	0,002212965
GATM_HUMAN	Glycineamidinotransferase, mitochondrial	1,695063349	0,012673881
CKAP4_HUMAN	Cytoskeleton-associatedprotein 4	1,601652224	0,000166712
CEL2A_HUMAN	Chymotrypsin-like elastase family member 2A	1,57034507	0,001435585
PSA5_HUMAN	Proteasomesubunitalpha type-5	1,568770938	0,002894956
KLK1_HUMAN	Kallikrein-1	1,46260278	0,033345907
LMNB2_HUMAN	Laminina-2B	1,436697709	0,011778001
E7EX73_HUMAN	Eukaryotic translation initiation factor 4 gamma 1	1,418576715	0,00128019
AMYP2A_HUMAN	Pancreaticalpha-amylase 2A	1,409056286	0,00904563
TYB4_HUMAN	Thymosin beta-4	1,34902955	0,021373347
AMRP_HUMAN	Alpha-2-macroglobulin receptor-associated p.	1,339554413	0,043633472
E9PK01_HUMAN	Elongationfactor 1-delta	1,299495585	0,002976972
CEL_HUMAN	Pancreaticlysophospholipase	1,287121057	0,005500881
PLEC_HUMAN	Plectin	1,202062102	0,003400044
LIPP_HUMAN	Pancreatictriacylglycerollipase	1,199448203	0,000837414
CYB5_HUMAN	Cytochrome b5	1,187106901	0,023347884
PRDX2_HUMAN	Peroxiredoxin-2	1,184190412	0,038073248
SE1L1_HUMAN	Protein sel-1 homolog 1	1,12927077	0,006274128
YBOX3_HUMAN	Y-box-bindingprotein 3	1,070276542	0,002278232
PIP_HUMAN	Prolactininducibleprotein	1,036996633	0,025382563
CBPA2_HUMAN	Carboxypeptidase A2	1,0354361	0,004918012
PDIA1_HUMAN	Proteindisulfide-isomerase	0,998406506	0,016945954
AHNK_HUMAN	Neuroblast differentiation-associated protein	0,931159249	0,001621318
A0A087X0X3_HUMAN	Heterogeneous nuclear ribonucleo-protein M	0,840340747	0,034232627
HNRPF_HUMAN	Heterogeneous nuclear ribonucleo-protein F	0,788528869	0,03024182
PPIB_HUMAN	Peptidyl-prolyl cis-trans isomerase B	0,788467472	0,005175838

HNRH1_HUMAN	Heterogeneous nuclear ribonucleo-protein H	0,780918986	0,001051223
NUDC_HUMAN	Nuclear migration protein nudC	0,780463175	0,049601681
VIME_HUMAN	Vimentin	0,779292891	0,003386928
YBOX1_HUMAN	Nuclease-sensitive element-binding protein 1	0,759930916	0,011660268
PDIA4_HUMAN	Proteindisulfide-isomerase A4	0,724169464	0,002196123
SND1_HUMAN	Staphylococcal nuclease domain-containing p.1	0,701575893	0,008457755
CBPA1_HUMAN	Carboxypeptidase A1	0,696649812	0,03164719
IMB1_HUMAN	Importinsubunit beta-1	0,625436604	0,006304313
RL13_HUMAN	60Sribosomial protein L13	0,622291069	0,023502963
SSRD_HUMAN	Translocon-associatedproteinsubunit delta	0,602829149	0,018241643
LA_HUMAN	Lupus La protein	0,599513229	0,027820277
PSB6_HUMAN	Proteasomesubunit beta type-6	0,594830733	0,041606267
C9J9K3_HUMAN	40S ribosomalprotein SA	0,547677265	0,047354331
LMNA_HUMAN	Prelamin A/C	0,533879772	0,027826684
D6R9P3_HUMAN	Heterogeneous nuclear ribonucleoprotein A/B	0,528721509	0,00880038
ML12A_HUMAN	Myosin regulatory light chain 12A	0,492587215	0,02228934
HSP7C_HUMAN	Heat shock cognate 71 kDa protein	0,4505999	0,031801407
ENPL_HUMAN	Endoplasmin	0,408208039	0,009900778
LMNB1_HUMAN	Laminin 1B	0,398168213	0,036108575
HNRPU_HUMAN	Heterogeneousnuclearribonucleoprotein U	0,377333781	0,029525001
AKA12_HUMAN	A-kinase anchor protein 12	0,367448186	0,044701593
SAHH_HUMAN	Adenosylhomocysteinase	0,346076082	0,043634514
CDC37_HUMAN	Hsp90 co-chaperone Cdc37	0,250956359	0,031565371

NGT: normal glucose tolerant subjects, IGT: impaired glucose tolerant subjects, p interaction between groups <0.05

Tab.S2 Correlations between HOMA-%beta and selected proteins.

	HOMA-%beta		
**Proteins**	**Pearson r**	**95% CI**	**P**
**14-3-3 T**	0.75	0.32 to 0.92	0.004
**SEL1L**	-0.81	-0.92 to 0.35	0.005
**PDIA1**	-0.51	-0.84 to 0.18	0.04
**PAK2**	0.75	0.21 to 0.93	0.02
**CEL2A**	-0.53	-0.85 to 0.13	0.04

Tab.S3 Correlations between Disposition index and selected proteins

	Disposition index		
**Proteins**	**Pearson r**	**95% CI**	**P**
**FUMH**	0.58	0.01 to 0.86	0.04
**ERO1B**	0.58	0.02 to 0.87	0.04
**CASPE**	-0.64	- 0.88 to - 0.10	0.02
**PAK2**	0.68	0.29 to 0.91	0.03
**CREB1**	0.58	0.02 to 0.87	0.04
**ARF1**	0.60	0.05 to 0.88	0.03
**SEL1L**	-0.71	-0.92 to 0.23	0.01

Figure S1. Gene ontology (GO) enrichment for differential genes between islets from subjects with high beta cells glucose sensitivity (representing in black bar) and islets from subjects with low beta cells glucose sensitivity (representing in grey bar). Categories were determined based on information provided by the online PANTHER classification system resource. All proteins were grouped based on biological process (panel A); molecular function (panel B); pathways analysis (panel C). Data are expressed as number of genes coding for the proteins identified in each group.

Fig.S1

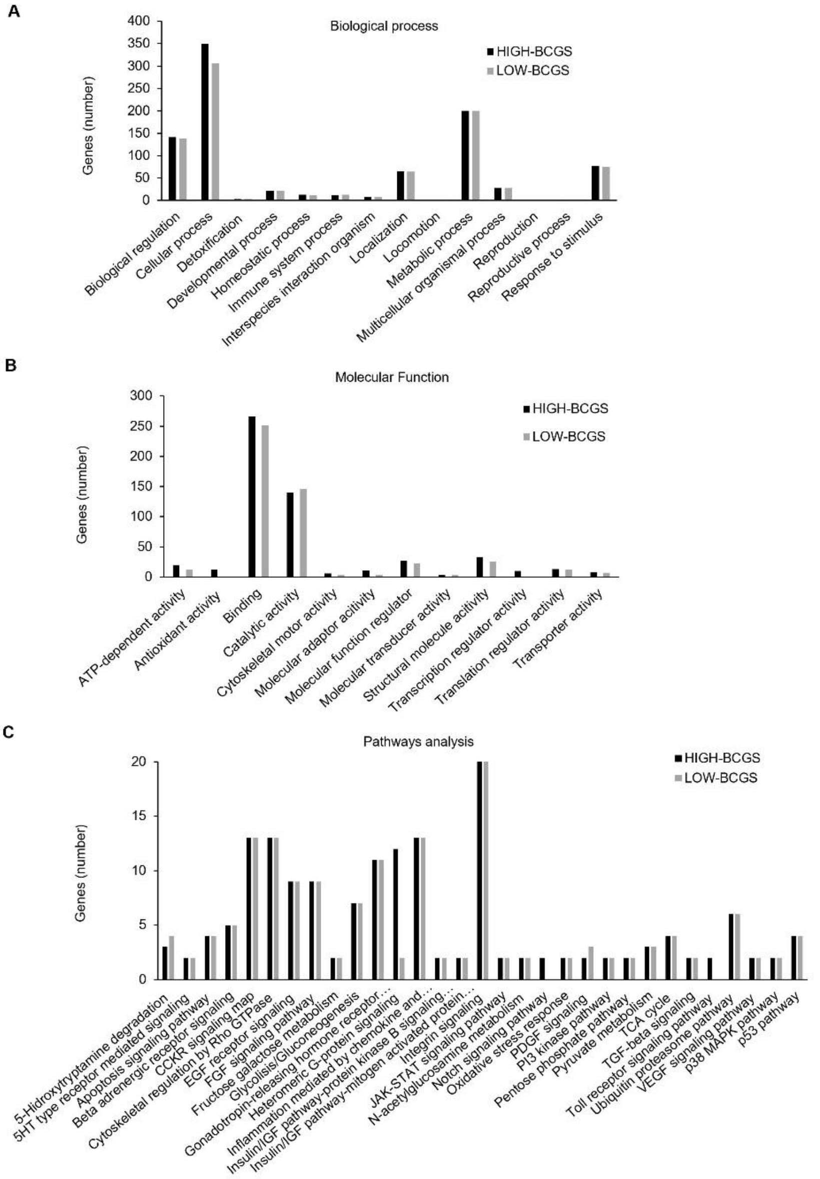

Fig.S2 **Differentially expressed proteins in IGT islets (black boxes) compared to NGT islets (grey boxes) after correcting for the expression of carboxypeptidase A1.** Proteins involved in the major detected molecular pathways are grouped according to their function: glucose metabolism (panel A), lipid metabolism (panel B), intracellular signaling pathways (panel C) insulin secretion and release (panel D); or apoptosis and proliferation (panel E). ***p<0.001; **p<0.01; *p<0.05; IGT: impaired glucose tolerant (n=5); NGT: normal glucose tolerant (n=7).

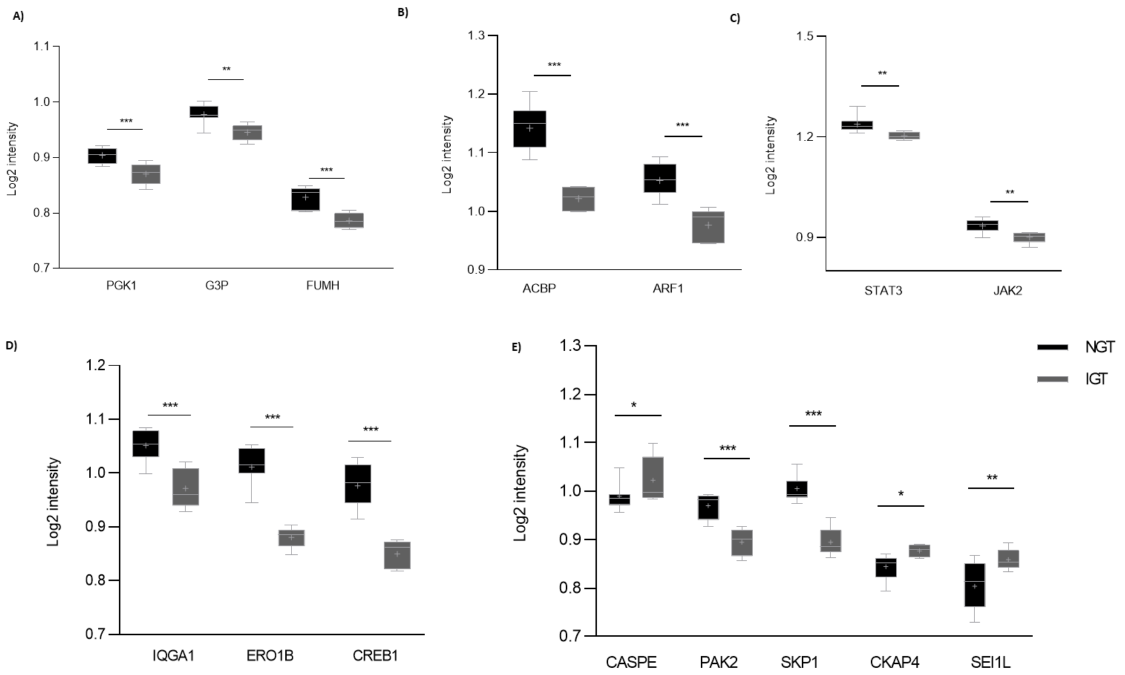

METHODS

Subjects and metabolic studies

Subjects, aged 18-75 years, without history of diabetes mellitus, candidates for pylorus-preserving pancreatoduodenectomy for extra-pancreatic tumors were recruited at the Digestive Surgery Unit and studied at the Centre for Endocrine and Metabolic Diseases unit (Agostino Gemelli University Hospital, Rome, Italy). The study protocol (ClinicalTrials.gov registration no. NCT02175459) was approved by the local ethics committee (P/656/CE2010 and 22573/14) (Rome, Italy) and all participants provided written informed consent prior any study procedure. In the preliminary visit, all participants underwent an anthropometric assessment (weight, BMI, waist and hips circumferences), pharmacological and pathological anamnesis was performed to note any current or previous therapies and comorbidities. Altered serum lipase and amylase levels prior to surgery, as well as morphologic criteria for pancreatitis, were considered exclusion criteria. Patients with severe obesity (BMI> 40), uncontrolled hypertension and/or diabetes (with HbA1c ≥ 58 mmol/mol (7.5%) and/or hypercholesterolemia were also excluded. Only patients with normal cardiopulmonary and renal function ascertained by medical history, physical examination, blood chemistry, electrocardiogram and urine analysis were subjected to a pre-surgery metabolic evaluation as follows:

1-Oral glucose tolerance test (OGTT). A standard 75 g oral glucose tolerance test was performed with measurement of glucose, insulin and C-peptide at 0, 30, 60, 90, 120 min after glucose load. Based on the pre-surgery OGTT results, we classified the patients according to the ADA classification (American Diabetes Association, 2019): subjects whose 2 h post glucose load was below 7.8 mmol/l were defined as normal glucose tolerant, subjects whose 2 h post glucose load was 7.8–10.9 mmol/l were defined as impaired glucose tolerant [1].

2-Euglycemic Hyperinsulinemic clamp for the evaluation of insulin sensitivity has been done after night fasting. During the test a fixed amount of insulin is infused (Actrapid HM, 40 mIU / m2 per min; Novo Nordisk, Copenhagen, Denmark) and at the same time a variable infusion of 20% glucose is started through an infusion pump. The glucose infusion rate is regulated on the basis of the glucose measured by capillary samples submitted every 5 minutes to maintain euglycemia with maximum variations of ± 5% with respect to the fasting glucose. The use of glucose is calculated during the last phase (30 min) of the clamp and is measured as an M value, i.e. mg of glucose metabolized per kg of body weight per minute.

3-Mixed Meal test. MMT was performed as previously described 17 (1-2). Patients were instructed to eat a meal of 830 kcal (107 kcal of protein, 353 kcal of fat and 360 kcal of carbohydrates) in 15 minutes. Blood samples were taken on an empty stomach and at 30 minutes intervals in the following 240 minutes (sampling time 0 ', 30', 60 ', 90', 120 ', 150', 180 ', 210' and 240 ') for measuring plasma concentrations of glucose, insulin, peptide C. Insulin levels were determined using a commercial RIA kit (Medical System, Immulite DPC, Los Angeles, CA). Plasma glucose concentrations were determined by the glucose oxidase technique, using a glucose analyser (Beckman Instruments, Palo Alto, CA, USA). Plasma C-peptide was measured by auto DELPHIA automatic fluoroimmunoassay (Wallac, Turku, Finland), with a detection limit of 17 pmol/L.

Calculations. During OGTT and MMT insulin secretion was derived from C-peptide levels by deconvolution. An estimate of the β cell function (defined as β-Cell Glucose Sensitivity) was obtained as an increase, compared to the basal value, of insulin secretion during the last 20 min of the test, divided by the corresponding increase in blood glucose as previously described [2]. Rate sensitivity, also estimated from OGTT modeling, is a β cell–functional parameter that represents the dependence of the ISR on the rate of change in glucose concentration and is related to early insulin release. Matsuda indexes [3] were calculated as indexes of whole-body insulin sensitivity based on insulin and glucose values obtained from the OGTT.

All metabolic evaluations were performed about a week before surgery and after a variable recovery period (assessed by the normalization of the inflammation indexes, weight stabilization, and absence of abnormal intestinal motility and deficiency of the exocrine pancreas symptoms) but before the start of any adjuvant antineoplastic treatment (chemotherapy or radiotherapy).

Surgical procedures

Indications for surgery were: periampullary tumors, pancreatic intraductal papillary tumors, mucinous cystic neoplasm of the pancreas, non-functional pancreatic neuroendocrine tumors. Pancreatoduodenectomy was performed according to the pylorus preserving technique. Briefly, the

pancreatic head, the entire duodenum, common bile duct, and gallbladder were removed en-bloc, leaving a functioning pylorus intact at the gastric outlet. All adjacent lymph nodes were carefully removed. The continuity of the gastrointestinal tract was restored by an end-to-side pancreatojejunostomy. Further downstream, an end-to-side hepaticojejunostomy and an end-to-side pylorojejunostomy were performed. The volume of pancreas removed during the surgery is constant (~50%), as previously reported by Schrader et al.[4]. This procedure guarantees repeatability of the model adopted and the possibility to compare samples from different subjects. A pancreatic sample was collected during the surgery, from the downstream edge of the surgical cut. Pancreas samples were frozen in liquid nitrogen and then stored at -80 until analysis.

Samples procession

Pancreatic tissue samples extracted during the surgery at A. Gemelli Hospital were placed in cryomolds, then included in OCT and quickly frozen in liquid nitrogen and stored - 80 ° C until sectioned, which took place at the Islet regeneration department at the Joslin Diabetes Center, Harvard Medical School (Boston-USA). The 8 µm sections were made using a cryostat at -20 °, then sections were fixed on slides at room temperature. We thus obtained about 40 slides per subject in study.

Subsequently, to avoid any contamination or chemical and physic stress on samples, the LCM (laser capture microdissection) was carried out on these sections for the islets' isolation as previously described in a methodological work [5]. To have sufficient material for mass spectrometry analysis, approximately 200 islets with similar size were collected per subject. The micro-dissected cells were therefore incubated with 15 µl of an elution / digestion buffer consisting of 8 mM $NH4HCO3$; 10 mM DTT; 50 Mm Trypsin pH 8 for 15 minutes at 37 ° C for protein digestion. The digestion was stopped by 0.1% TFA. All peptide samples were dried down in Speed Vac remove TFE, and resuspended in 20 mM $NH4HCO3$ for LC-MS/MS analysis.

Proteomic analysis

The proteomics analysis was performed at the Pacific Northwest laboratories of Richland (WA). The use of the high-performance liquid chromatography-mass spectrometry (HPLC-MS) technique guarantees the identification of multiple peptide's sequences recognizable with high confidence in complex samples and the comparison of different samples with a similar protein profile.

LC-MS/MS analyses were performed on a custom-built automated LC system coupled on-line to an LTQ-Orbitrap mass spectrometer (Thermo Scientific, San Jose, CA) via a nano-electrospray ionization interface as previously described [6]. Briefly, 0.75 µg of peptides were loaded onto long reversed-phase capillary columns with 75-µm-inner diameter packed using 3 µm Jupiter C18 particles (Phenomenex, Torrance, CA). The mobile phase was held at 100% A (0.1% formic acid) for 20 min, followed by a linear gradient from 0 to 60% buffer B (0.1% formic acid in 90% acetonitrile) over 85 min. The instrument was operated in data-dependent mode with an m/z range of 400–2000, in which a full MS scan with a resolution of 100K was followed by 6 MS/MS scans. The 6 most intensive precursor ions were dynamically selected in the order of highest intensity to lowest intensity and subjected to collision-induced dissociation using a normalized collision energy setting of 35% and a dynamic exclusion duration of 1 min. The heated capillary was maintained at 200 °C, while the ESI voltage was kept at 2.2 kV.

LC-MS/MS raw data were converted into data files using Extract_MSn (version 3.0) in Bioworks Cluster 3.2 (Thermo Fisher Scientific, Cambridge, MA). MS Generating-Function (MSGF) scores were generated for each identified spectrum as described previously by computing rigorous p-values [7]. Fully tryptic peptides with MSGF score <5E-10 and mass measurement errors <3 ppm were accepted as identifications. All peptides that passed the filtering criteria were input into the Protein Prophet program [8] to generate a final non-redundant list of proteins. Label-free MS intensity-based quantification was performed using the accurate mass and time (AMT) tag approach as previously described [9]. The analysis of the raw data was performed through a client-server application (Proteome Discovered version 2.0 produced by Thermo Fisher) able to identify different proteins by comparing the mass spectra of the digested fragments with the information contained in a selected FASTA database. Through the UniProt service, which collects information from the most used Swiss-prot, TrEMBL and PIR databases [10], we clarified the function and characteristics of each protein. Then using PANTHER classification software (Protein Analysis Through Evolutionary Relationships) it was possible to classify the proteins (and their coding genes) expressed in each single group under study based on their biological functions and molecular function [11].

Tab. S4 List of Antibodies with Corresponding Dilutions and Antigen Retrieval Buffers

Order	Antibody	Supplier	Clone	Catalog	Diluition Facor	Opal Pairing	Retrival and Incubation Time
1	IQ-GAP1	Abcam	[EPR5220]	133490	1:100	520	ER1 30'
2	SEL1L	Invitrogen	polyclonal	PA5-88333	1:300	690	ER1 40'
3	GLUCAGON	Santa Cruz	(C11)	sc-514592	1:500	570	ER1 30'
4	INSULIN	Invitrogen	polyclonal	PA5-120784	1:600	480	ER1 30'

Bibliography:

1. American Diabetes, A., *2. Classification and Diagnosis of Diabetes: Standards of Medical Care in Diabetes-2019.* Diabetes Care, 2019. **42**(Suppl 1): p. S13-S28.
2. Mezza, T., et al., *Pancreaticoduodenectomy model demonstrates a fundamental role of dysfunctional beta cells in predicting diabetes.* J Clin Invest, 2021. **131**(12).
3. Matsuda, M. and R.A. DeFronzo, *Insulin sensitivity indices obtained from oral glucose tolerance testing: comparison with the euglycemic insulin clamp.* Diabetes Care, 1999. **22**(9): p. 1462-70.
4. Menge, B.A., et al., *Metabolic consequences of a 50% partial pancreatectomy in humans.* Diabetologia, 2009. **52**(2): p. 306-17.
5. Cefalo, C.M.A., et al., *A Systematic Comparison of Protocols for Recovery of High-Quality RNA from Human Islets Extracted by Laser Capture Microdissection.* Biomolecules, 2021. **11**(5).
6. Zhou, J.Y., et al., *Improved LC-MS/MS spectral counting statistics by recovering low-scoring spectra matched to confidently identified peptide sequences.* J Proteome Res, 2010. **9**(11): p. 5698-704.
7. Kim, S., N. Gupta, and P.A. Pevzner, *Spectral probabilities and generating functions of tandem mass spectra: a strike against decoy databases.* J Proteome Res, 2008. **7**(8): p. 3354-63.
8. Nesvizhskii, A.I., et al., *A statistical model for identifying proteins by tandem mass spectrometry.* Anal Chem, 2003. **75**(17): p. 4646-58.
9. Qian, W.J., et al., *Large-scale multiplexed quantitative discovery proteomics enabled by the use of an (18)O-labeled "universal" reference sample.* J Proteome Res, 2009. **8**(1): p. 290-9.
10. UniProt, C., *UniProt: the Universal Protein Knowledgebase in 2023.* Nucleic Acids Res, 2023. **51**(D1): p. D523-D531.
11. Thomas, P.D., et al., *PANTHER: Making genome-scale phylogenetics accessible to all.* Protein Sci, 2022. **31**(1): p. 8-22.
